# Unveiling the hidden burden of COVID-19 in Brazil’s obstetric population with severe acute respiratory syndrome: A machine learning model

**DOI:** 10.1371/journal.pone.0330375

**Published:** 2025-08-22

**Authors:** Veridiana Freire Franco, Tatiana Assunção Zaccara, Ornella Scardua Ferreira, Rafaela Alkmin da Costa, Agatha Sacramento Rodrigues, Rossana Pulcinelli Francisco

**Affiliations:** 1 Departamento de Obstetricia e Ginecologia da Faculdade de Medicina da Universidade de Sao Paulo, Sao Paulo, Brazil; 2 Divisão de Clínica Obstétrica do Hospital das Clínicas da Faculdade de Medicina da Universidade de São Paulo, Sao Paulo, Brazil; 3 Departamento de Estatistica da Universidade Federal do Espirito Santo, Vitoria, Espírito Santo, Brazil; Fundacao Oswaldo Cruz Instituto Rene Rachou, BRAZIL

## Abstract

**Objective:**

To predict the actual number of COVID-19 cases in Brazilian pregnant and postpartum women diagnosed with Severe Acute Respiratory Syndrome using a predictive model created based on data from Brazilian database.

**Methods:**

This is a cross-sectional study with pregnant and postpartum women diagnosed with Severe Acute Respiratory Syndrome (SARS) between January 2016 and November 2021 in Brazil. Patients were divided into two groups (COVID-19 and non-COVID-19) for comparative analysis, and a predictive model was constructed to classify cases without a defined causative agent.

**Main outcome measures:**

Estimated number of COVID cases in obstetric patients with SARS and no defined agent.

**Results:**

38,774 pregnant and postpartum women diagnosed with SARS were identified and categorized by date and causative agent. Women in the COVID-19 group (19.138) were older (29.86 ± 7.20 years), self-reported more frequently as non-white race (50.9%), and more often had educational status marked as blank or ignored (29.6% and 26.9%, respectively) compared to the Other confirmed agents’ group (2.233). The groups differed in all presented variables, and patients in the COVID-19 group were diagnosed more often in the third trimester of pregnancy or in the postpartum period. Using the XGBoost model, 13,978 cases of SARS with undefined etiology from 2020 and 2021 were reclassified: 13,799 (98.7%) as predicted COVID-19 and 179 (1.3%) as predicted non-COVID-19.

**Conclusions:**

The number of COVID-19 cases and deaths in the obstetric population were even higher than reported by authorities, indicating a significant impact on the maternal mortality ratio during this period.

## Introduction

The COVID-19 pandemic highlighted the precarious state of Brazil’s healthcare system, revealing difficulties in accessing care and inequalities in services across the different regions [1]. The Coronavirus Disease 2019 (COVID-19) is a syndrome caused by the new coronavirus (SARS-CoV-2, Severe Acute Respiratory Syndrome Coronavirus 2), an RNA virus (Ribonucleic Acid) that has been known since the 1960s and which classically affects the human respiratory tract [2] The outbreak began in the city of Wuhan, China, at the end of December 2019, and it is suspected that the first transmissions occurred from animals to humans, before transmission between humans.

On March 11, 2020, COVID-19 was characterized by the WHO as a pandemic, and, as of early March 2023, more than 600 million cases of COVID-19 and more than 6 million deaths have been confirmed worldwide [3]. Transmission occurs through direct contact (within one meter) and/or indirect contact (via fomites) with infected individuals, facilitated by saliva and respiratory secretions.

The clinical manifestation of the disease can be diverse, ranging from asymptomatic forms to a broad spectrum of respiratory or gastrointestinal symptoms, and can reach severe and potentially fatal conditions. The most frequently reported symptoms are fever, cough, fatigue, and shortness of breath, which are similar to the symptoms of other viral respiratory infections, such as the seasonal flu, and tend to appear more commonly between the fourth and fifth day of exposure. However, studies show that the incubation period of the virus can last up to 14 days. [4] Anosmia (loss of smell) and ageusia (loss of taste) have also been frequently reported symptoms in COVID-19 patients and appear to precede other symptoms.

The overall mortality of COVID-19 is variable, and can be less than 5% in patients under the age of 40 and as high as 60% in those aged between 80 and 89. Another critical factor influencing the hospital mortality rate is whether an ICU admission is required [5]. It is also important to note that since not all the people who died during the pandemic were tested for the presence of SARS-CoV-2, the actual number of deaths from COVID-19 may be much higher than reported.

The elderly, immunosuppressed patients, and those with comorbidities (systemic arterial hypertension, diabetes mellitus, and obesity) are considered to be at risk of the most severe forms of the disease. Initially, pregnant and puerperal women were not considered to be at high risk of the severe form of the disease [6]. Although the presence of severe complications associated with COVID-19 in young women was low, studies have shown that pregnant and puerperal women had a significantly higher chance of ICU admission, the need for invasive ventilation, the use of ECMO (Extracorporeal Membrane Oxygenation) and death when compared to women in the same age group who were not pregnant [7,8].

The lack of diagnostic tests and access to the hospital, especially at the beginning of the pandemic, resulted in a high rate of underreporting of COVID-19 cases and related deaths [9]. The quality of mandatory disease notification is a global issue, leading to difficulties in identifying the epidemiological reality of a given geographic area. This can hinder the planning and implementation of collective control measures [10].

The underreporting of COVID-19 deaths was a worldwide problem. The estimated notification rate (number of confirmed cases reported by the Ministry of Health (MoH) divided by the number of expected cases estimated from the number of deaths) in Brazil, up to April 2020, was 9.2% [11], suggesting that less than 10% of COVID-19 cases were diagnosed and that the notification of confirmed cases was far below other countries such as the USA, Spain or Italy, whose notification rates are higher than 15%, or South Korea and Germany, where more than 50% of cases were diagnosed and notified [12]. A study published in March 2022 indicated that COVID-19 deaths could be three times higher than the official reports. In 2020, in Brazil, the underestimated death rate caused by the coronavirus reached 18% [13].

At the onset of the pandemic, the majority of COVID-19 cases in pregnant and postpartum women were classified as mild or moderate, with only a small proportion requiring intensive care or mechanical ventilation [14–16]. However, the literature has shown that the obstetric group had an increased risk of serious complications, such as the need for ICU admission, the use of mechanical ventilation, and death when compared to the group of non-pregnant women [15,17–19].

It is well known that pregnant women are more susceptible to severe respiratory infections. In an epidemiological bulletin released by the Ministry of Health in May 2020, 252 pregnant women had SARS, of which 36 died, with COVID-19 as the confirmed cause of death [20], making the country the first in maternal deaths caused by infection with the new coronavirus [11]. Four months after the first case of COVID-19 infection in the country was reported, the number of maternal deaths reached 124 (12.7% of the hospitalized obstetric population) [21]. According to the Brazilian Obstetric Observatory COVID-19 (OOBr COVID-19), maternal deaths in the first five months of 2021 (a weekly average of 47.9 deaths) exceeded the number reported in 2020 (a weekly average of 12.1 deaths) [22].

In addition to the maternal immune system’s reduced effectiveness due to tolerance to antigens expressed by fetal cells [23], the greater vascularization of the nasopharyngeal epithelium, which is swollen with increased levels of progesterone, can also facilitate the pathogen’s entry. In addition, other adaptive functional and anatomical changes can contribute to making pregnant women more intolerant of hypoxia, such as [15] elevation of the diaphragm by the gravid uterus, causing a decrease in functional residual capacity of around 10–25%; increased respiratory effort caused by the stimulating effect of progesterone; increased microvascular pressure and pulmonary capillary permeability, increasing the risk of developing SARI; reduced vascular resistance and plasma colloid pressure, increasing the risk of acute pulmonary edema.

Analyzing the sociodemographic risk factors associated with adverse outcomes among obstetric cases, it has been shown that advanced maternal age (between 35–44 years) is a risk factor in this group, with pregnant women in this age group almost four times more likely to need OI and twice as likely to die when compared to non-pregnant women [7].

Variables related to social vulnerability, such as living in a peri-urban area, increased the risk of an adverse COVID-19 outcome more than threefold. The risk of death and worse clinical conditions at the time of hospital admission was higher in black pregnant and postpartum women [24]. Existing barriers, such as the difficulty in accessing prenatal and postpartum care in Brazil, have been further exacerbated during the pandemic.

Regarding the time of pregnancy (non-obstetric, pregnant, and postpartum), postpartum was associated with a higher risk of ICU admission, need for mechanical ventilation, and death when compared to pregnant women and the non-obstetric population [25].

Among the clinical characteristics that can be considered risk factors for a worse outcome among pregnant and postpartum women with COVID-19, obesity and gestational diabetes were the most commonly reported comorbidities, with obesity presenting a risk of death twice as high among infected pregnant women when compared to those who survived [26].

Obstetric adverse events such as gestational diabetes, pre-eclampsia, prematurity, low birth weight, and fetal death are also reported in the literature with a greater chance of occurring in pregnant women with COVID-19, especially in those who develop the severe forms of the disease [15,27–29].

Maternal mortality rates from COVID-19 in high-income countries were lower when compared to rates in low- and middle-income countries, which were up to six times higher [30].It is important to note that maternal mortality is directly linked to the care provided during pregnancy and the puerperium. The suspension of prenatal appointments and tests during the pandemic, along with limited access to diagnostic tests and inadequate care and support services, may be directly related to the scenario presented in Brazil.

In 2021, the maternal mortality ratio in Brazil reached 107.53 deaths per 100,000 live births. When compared to previous years, the ratio was 55.31 deaths per 100,000 live births in 2019 and 71.97 deaths per 100,000 live births in 2020 [31]. According to data obtained by the Brazilian Obstetric Observatory (OOBr), the total number of maternal deaths increased by 77% between 2019 and 2021 [22]. In addition to the lack of trained professionals to care for pregnant and postpartum women, the limited number of ICU beds for the obstetric population and their unequal distribution among Brazilian regions may have contributed to the increase in maternal mortality during this period.

However, even considering the initial difficulties, with the gradual availability of tests and a greater understanding of the disease, establishing a diagnosis of COVID-19, especially in the group of pregnant and postpartum women (a highly vulnerable group), has become of great importance not only for managing cases, but also as a tool for assessing the quality of care provided to this population. The predictive model of the actual number of COVID-19 cases is not available for pregnant and postpartum women [32], and the national and worldwide impact of the pandemic on this subpopulation remains unknown.

Thus, one way to estimate the actual number of cases is to utilize machine learning (ML) tools to correct for possible underreporting by reclassifying cases without a defined etiology. This tool is part of a subfield of artificial intelligence that involves building algorithms and models capable of learning from data. Instead of being explicitly programmed to perform a task, these models are trained on a dataset to identify patterns and make predictions or decisions based on new data [33]. In the health field, supervised machine learning algorithms have already been used as a diagnostic aid in various areas [34–36].

This study aimed to create a predictive model based on data from Brazilian pregnant and postpartum women who had severe acute respiratory syndrome (SARS) confirmed either by COVID-19 or other etiological agents. The model was applied to the reported SARS cases in which the etiological agent was marked as “Unspecified,” “Blank,” or “Ignored” to estimate the number of cases that were likely caused by COVID-19 in this population.

## Materials and methods

This is a cross-sectional analysis with a retrospective review of data obtained from the Influenza Epidemiological Surveillance Information System (SIVEP-Gripe). The SIVEP-Gripe database contains records of hospitalized cases and deaths from SARS. In Brazil, notification is mandatory for influenza-like illness, characterized by at least two of the following symptoms: fever, chills, sore throat, headache, cough, runny nose, olfactory or taste disorders along with dyspnea or respiratory discomfort, persistent chest pressure, oxygen saturation (O_2_) below 95% in room air, or bluish lips or face.

We selected cases of pregnant and postpartum women aged between 10 and 55 with a diagnosis of SARS according to the SIVEP-Gripe definition, with or without a defined etiological diagnosis, from 2016 to 2021. The data were extracted on April 4, 2024, from the official website of the Ministry of Health of Brazil—DATASUS [37].

This study used R software (version 4.3.3) and the RStudio integrated development environment to process the data and perform the statistical analyses. Both the data and analyses can be accessed in the GitHub repository at https://github.com/observatorioobstetrico/covid19_vs_unspec.

### Predictive model for classifying cases with undefined etiological agent as COVID-19 or non-COVID-19

Before constructing the predictive model for the presence or absence of a COVID-19 diagnosis, it was necessary to understand the etiological agents causing SARS in pregnant and postpartum women. Upon analyzing cases from 2020 and 2021, few non-COVID-19 cases with defined etiological diagnoses were found. Therefore, data from previous years were included, starting from January 1, 2016, when most variables were consistent across all selected years.

In this study, the following variables were used: age (in years); ethnicity (categories: white, non-white, blank, and ignored); education level (categories: up to elementary, high school, higher education, blank, and ignored); gestational stage (categories: first trimester, second trimester, third trimester, postpartum, and ignored); influenza vaccination, fever, cough, sore throat, dyspnea, respiratory discomfort, O_2_ saturation below 95%, diarrhea, heart disease, chronic lung disease, chronic kidney disease, obesity (all with categories: yes, no, blank, and ignored), and the number of SARS notifications on the patient’s admission date in a health service (created from the variable related to the date of the first symptoms). Notably, outcome variables (Intensive Care Unit (ICU) requirement and ventilatory support) were not considered, as they did not add additional information to the predictive models. Variables not present in any of the periods (e.g., anosmia, unavailable before 2020) were not considered.

Two groups were established for the predictive model

-COVID-19 Group: SARS cases in pregnant and postpartum women from January 2020 until November 2021, with a confirmed COVID-19 diagnosis.
-Non-COVID-19 Group: SARS cases in pregnant and postpartum women from January 2016 to November 2021, with a confirmed diagnosis of another etiological agent.

A comparative analysis between the two groups was performed using t-tests (aided by Cohen’s d statistic for effect size), Pearson’s Chi-square (aided by Cramér’s V statistic to evaluate the association’s effect size), and Fisher’s Exact test when appropriate.

### Modeling and sampling

Twelve models were adjusted using the following techniques: Linear Discriminant Analysis, Quadratic Discriminant Analysis, Classification Trees, Bagging, Random Forests, K-Nearest Neighbors, Logistic Regression, Lasso Logistic Regression, RBF SVM, Linear SVM, Polynomial SVM, and XGBoost [38].

The data set was divided into training and test samples: 75% of the data was used for training and 25% of the data was used to test the models’ performance and prediction on new data. From the training sample, K-Fold cross-validation, a resampling technique that randomly divides the data into k groups of approximately equal size, was used to select the best hyperparameter configurations. Under this approach, k = 10 and 3 repetitions were considered. Additionally, the SMOTE resampling technique [39] was adopted. SMOTE creates synthetic data from the minority class using the K-Nearest Neighbors method to correct a moderate imbalance between categories (the non-COVID-19 group was about 10% of the total available data). Therefore, k = 5 and an over ratio = 0.5 were used, meaning the non-COVID-19 category was increased to 50% of the COVID-19 category.

### Interpretability

The best-performing model was XGBoost, a non-interpretable model (black box) [40]. This means it is impossible to directly understand the relationship between the covariates and the response variable. Thus, interpretability measures are needed to understand the reasons influencing case classifications, either COVID-19 or non-COVID-19. One such measure is SHAP (SHapley Additive exPlanations) [41], which measures the contribution of covariates in the model using all possible combinations of the presence and absence of the covariates. SHAP can explain the model’s decisions individually and globally, and in this study, its global interpretation was considered (interpretation based on the average results of all outcomes). The contribution is considered positive when the variable increases the chance of the outcome of interest occurring (in our case, diagnosing COVID-19) and negative when the variable decreases the possibility of such an outcome.

### Classification of cases with undefined etiological agent

The XGBoost model was then applied to data from 2020 to 2021 for SARS cases in pregnant and postpartum women where the etiological agent was marked as “Unspecified,” “Ignored,” or “Blank.” The COVID-19 cases rate (the number of cases per 100,000 live births) and the maternal mortality ratio (the number of maternal deaths per 100,000 live births) were also estimated, considering both diagnosed and predicted COVID-19 cases.

## Results and discussion

**Fig 1:**
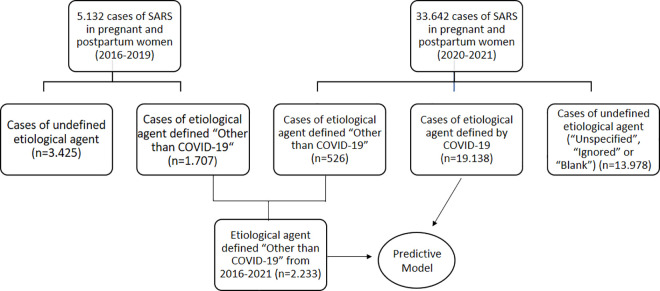
Flowchart of case selection for the predictive model’s construction.

In the present study, 38,774 cases of SARS in pregnant and postpartum women between January 2016 and November 2021 were identified. These cases were classified into two groups: SARS in pregnant and postpartum women from 2016–2019 (5,132 cases, 13·2%) and SARS in pregnant and postpartum women from 2020–2021 (33,642 cases, 86·8%). Subsequently, both groups were subdivided according to the etiological agent: SARS with a defined non-COVID-19 etiological agent, SARS with a defined COVID-19 etiological agent, and SARS with an undefined etiological agent (“Unspecified,” “Ignored,” or “Blank”). A comparative analysis was then performed between the groups of SARS confirmed by COVID-19 or Other confirmed etiological agents (2016–2021) (Fig 1).

The groups differed in number of SARS notifications on admission’s date, with 47·6 ± 25·89 notifications in the COVID-19 group and 9·41 ± 14·36 notifications in the non-COVID-19 group (Other confirmed agents).

Regarding to demographic data, women in the COVID-19 group were older (29·86 ± 7·20 vs. 27·45 ± 7·05 years, p < 0·001), identified more frequently as non-white (50·9% vs. 43·9%, p < 0·001), and more often had their educational level listed as blank or ignored (29·6% and 26·9%, vs. 13·5% and 24·1%, respectively, p < 0·001) compared to women in the non-COVID-19 group.

All variables evaluated showed statistical differences between groups, and patients in the COVID-19 group were diagnosed more often in the third trimester of pregnancy or in the postpartum period. Table 1 shows the comparative analysis data and the effect size for each variable.

**Table 1:** Sociodemographic Characteristics of Pregnant and Postpartum Women with SARS Confirmed by COVID-19 or Other Confirmed Agents.

Characteristics	COVID-19	Other confirmed agents	p	Effect size*
SARS notifications on the admission day; mean ± sd	47·60 ± 25.89	9·41 ± 14·36	< 0·001	1·82
Age (years); mean ± sd	29·86 ± 7·20	27·45 ± 7·05	< 0·001	0·34
Race, N (%)			< 0·001	0·08
White	6649 (34·7%)	1011 (45·3%)		
Non-white	9749 (50·9%)	981 (43,9%)		
Blank	262 (1·4%)	52 (2·3%)		
Ignored	2478 (12·9%)	189 (8·5%)		
Education level, N (%)			< 0·001	0·13
Up to elementary	2317 (12·1%)	434 (19·4%)		
High school	44445 (23·2%)	715 (32·0%)		
Higher education	1557 (8·1%)	245 (11·0%)		
Blank	5664 (29·6%)	301 (13·5%)		
Ignored	5155 (26·9%)	538 (24·1%)		
Influenza vaccination, N (%)			< 0·001	0·21
Yes	2781 (14·5%)	609 (27·3%)		
No	5996 (31·3%)	1156(51·8%)		
Blank	3850 (20·1%)	124 (5·6%)		
Ignored	6511 (34·0%)	344 (15·4%)		
Gestational stage, N (%)			< 0·001	0·15
First trimester	1394 (7·3%)	366 (16·4%)		
Second trimester	4014 (21·0%)	701 (31·4%)		
Third trimester	9410 (49·2%)	920 (41·2%)		
Postpartum	3504 (18·5%)	204 (9·1%)		
Ignored	780 (4·1%)	42 (1·9%)		

COVID-19: Coronavirus Disease 2019; sd: standard deviation; N: number; p: p-value; %: percentage·

* Effect size given by Cramer’s V, except in the Age variable, where Cohen’s C was used

Moreover, there was a significant difference between groups concerning the distribution of symptoms, the presence of comorbidities, and the completion of these fields in the SIVEP-Gripe form (Table 2). Considering only fields answered as “Yes” and “No,” the frequency of sore throat and diarrhea was higher in the COVID-19 group. In contrast, fever, cough, and respiratory discomfort were more common in the group with other confirmed agents. The frequency of dyspnea and O2 saturation < 95% were similar in both groups. Similarly, when comparing the frequency of completed responses about the presence of comorbidities, heart disease, chronic kidney disease, and obesity were also more frequent in the COVID-19 group than in the Other confirmed agents’ group. Conversely, the frequency of chronic lung disease was higher in the non-COVID-19 group.

**Table 2:** Clinical presentation and presence of comorbidities of influenza-like illnesses in pregnant and postpartum women with SARS confirmed by COVID-19 and by Other confirmed agents.

Symptom, N (%)	COVID-19	Other confirmed agents	p	Effect size*
Fever			< 0·001	0·18
Yes	10129 (52·9%)	1803 (80·7%)		
No	6318 (33·0%)	378 (16·9%)		
Blank	2519 (13·2%)	38 (1·7%)		
Ignored	172 (0·9%)	14 (0·6%)		
Cough			< 0·001	0·16
Yes	12882 (67·3%)	2022 (90·6%)		
No	4273 (22·3%)	173 (7·7%)		
Blank	1834 (9·6%)	32 (1·4%)		
Ignored	145 (0·8%)	6 (0·3%)		
Sore throat			< 0·001	0·19
Yes	3917 (20·5)	913 (40·9%)		
No	10683 (55·8%)	1191 (53·3%)		
Blank	4251 (22·2%)	77 (3·4%)		
Ignored	287 (1·5%)	52 (2·3%)		
Dyspnea			< 0·001	0·10
Yes	10721 (56·0%)	1438 (64·4%)		
No	5884 (30·7%)	729 (32·2%)		
Blank	2391 (12·5%)	56 (2·5%)		
Ignored	140 (0·7%)	20 (0·9%)		
Respiratory discomfort			< 0·001	0·16
Yes	8424 (44·0%)	1495 (67·0%)		
No	7267 (38·0%)	643 (28·8%)		
Blank	3239 (16·9%)	69 (3·1%)		
Ignored	208 (1·1%)	26 (1·2%)		
O_2_ saturation < 95%			< 0·001	0·16
Yes	7187 (37·6%)	675 (30·2%)		
No	8315 (43·4%)	1388 (62·2%)		
Blank	3391 (17·7%)	93 (4·2%)		
Ignored	245 (1·3%)	77 (3·4%)		
Diarrhea			<0·001	0·25
Yes	1847 (9·7%)	54 (2·4%)		
No	12270 (64·1%)	784 (35·1%)		
Blank	4746 (24·8%)	1369 (61·3%)		
Ignored	275 (1·4%)	26 (1·2%)		
Heart disease			< 0·001	0·25
Yes	1157 (6·0%)	54 (2·4%)		
No	5945 (31·1%)	1517 (68·4%)		
Blank	11899 (62·2%)	617 (27·6%)		
Ignored	137 (0·7%)	45 (2·0%)		
Chronic lung disease			< 0·001	0·26
Yes	117 (0·6%)	115 (5·1%)		
No	6660 (34·8%)	1452 (65·0%)		
Blank	12212 (63·8%)	620 (27·8%)		
Ignored	149 (0·8%)	46 (2·1%)		
Chronic kidney disease			< 0·001	0·23
Yes	124 (0·6%)	8 (0·4%)		
No	6607 (34·5%)	1553 (69·5%)		
Blank	12263 (64·1%)	627 (28·1%)		
Ignored	144 (0·8%)	45 (2·0%)		
Obesity			< 0·001	0·24
Yes	1183 (6·2%)	61 (2·7%)		
No	5868 (30·7%)	1489 (66·7%)		
Blank	11922 (62·3%)	639 (28·6%)		
Ignored	165 (0·9%)	44 (2·0%)		

COVID-19: Coronavirus Disease 2019; N: number; p: p-value; %: percentage

* Effect size given by Cramer’s V

Among the twelve models evaluated, the XGBoost model demonstrated the highest accuracy (Table 3), highlighting its high performance in correctly predicting COVID-19 and non-COVID-19 cases. For this reason, this model was chosen to classify cases without a confirmed etiological agent. The hyperparameters of this model are shown in Table 4 – the hyperparameters considered in the other models can be accessed in S1 Table (supplementary table).

**Table 3:** Performance of the tested models to classify cases where the etiological agent was confirmed as “COVID-19” or “Other confirmed agents”.

Model	Accuracy	Sensitivity	Specificity	PPV	NPV
XGBoost	97·42%	99·58%	78·70%	97·59%	95·61%
Linear Discriminant Analysis	94·39%	98·75%	56·68%	95·17%	83·96%
Quadratic Discriminant Analysis	75·20%	74·42%	81·95%	97·27%	27·04%
Classification Trees	97·30%	99·27%	80·32%	97·76%	92·71%
Bagging	97·19%	99·27%	79·24%	97·64%	92·62%
Random Forest	97·36%	99·69%	77·26%	97·43%	96·61%
K-nearest Neighbors	95·13%	99·48%	57·58%	95·30%	92·73%
Logistic Regression	96·61%	99·33%	73·10%	96·96%	92·68%
Lasso Logistic Regression	96·54%	99·31%	72·56%	96·90%	92·41%
RBF SVM	96·87%	99·10%	77·62%	97·45%	90·91%
Linear SVM	96·37%	99·69%	67·69%	96·39%	96·15%
Polynomial SVM	96·35%	99·69%	67·51%	96·37%	96·14%

*RBF: Radial Basis Function; SVM: Support Vector Machine;* PPV: positive predictive value; NPV: negative predictive value.

**Table 4:** Hyperparameters adopted in the XGBoost model.

Hiperparameters	Value
trees	1000·00000
try	16·00000
min_n	11·00000
tree_depth	9·00000
learn_rate	0·07760
loss_reduction	0·00157
sample_size	0·22400

To interpret the proposed XGBoost model’s results globally, we used the SHAP summary plot to visualize the importance and relationships of each variable with the predicted outcomes.

In this plot, the input variables are ordered on the y-axis in descending order according to the average absolute SHAP values. Each of these variables represents an observation, and the points are distributed horizontally along the x-axis according to their respective SHAP value: when the density of the SHAP value is high, the points spread vertically. The color bar represents the raw values of the variables for each point on the plot: if a particular variable’s value is high, the point is colored in various shades of red; if the value is low, the point is colored in multiple shades of blue. The color distribution horizontally allows us to naturally understand the overall relationship of each variable with the SHAP values.

**Fig 2:**
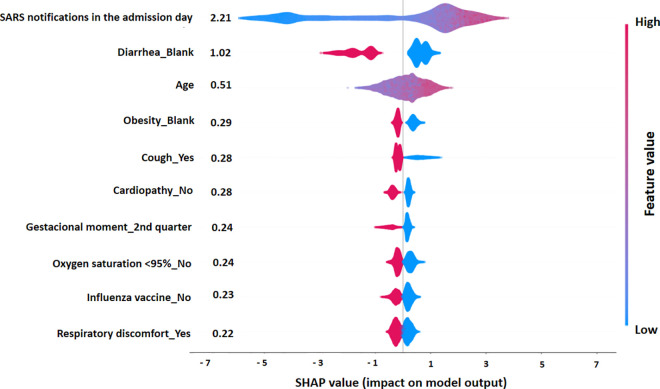
SHAP Summary Plot, ranked by the mean absolute SHAP values, for the XGBoost model.

In Fig 2, the low values of SARS notifications on the patient’s admission date have negative SHAP values (points elongate to the left with increasingly blue tones), and high values of SARS notifications on the patient’s admission have positive SHAP values (points extend from zero to the right with more purplish and reddish tones). This means that pregnant and postpartum women who reported the first symptoms on a day with a high number of SARS notifications belonged to the COVID-19 group. Conversely, being in the 2nd trimester of pregnancy and being marked as “Blank” in the SIVEP-Gripe form fields for diarrhea and obesity, as well as marking “Yes” in the fields for cough and respiratory discomfort and “No” in the fields for heart disease, O_2_ saturation less than 95%, and Influenza vaccination, determined the prediction of SARS by other etiological agents. Age did not influence the model’s classification, as evidenced by the point cloud with an apparent mixture of colors.

Analyzing the summary plot of the average contribution of each variable, given by the mean of the absolute SHAP value, the result is expressed in an intuitively interpretable unit by taking the mean of the absolute SHAP value. In the plot, the contributions are represented by the values in the boxes on the left, and the variables with the highest contributions impact the model’s decisions more. The quantity of daily SARS notifications was the most influential variable, changing the predicted probability of COVID-19 diagnosis by 2·21 percentage points on average. The opposite is seen when evaluating the variables Influenza vaccination (category: “No”) and respiratory discomfort (category: “Yes”), as they were the least informative variables.

**Fig 3:**
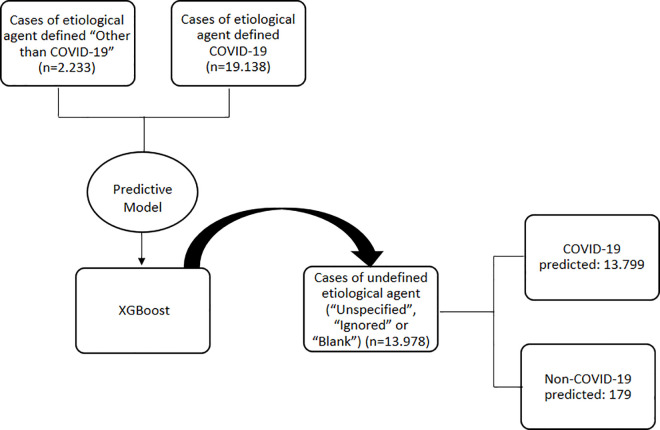
Flowchart of patients reclassified after the XGBoost model was applied.

After defining the most accurate prediction model, the cases without defined etiology during the study period were revisited 13,978 cases of SARS in pregnant and postpartum women between 2020 and 2021 with undefined etiology (“Unspecified,” “Ignored,” or “Blank”) were identified. These cases were reclassified into two groups using the model previously mentioned: predicted COVID-19 (13,799; 98·7%) and predicted non-COVID-19 (179; 1·3%) (Fig 3). The sociodemographic characteristics of these groups are shown in S1 Table. These predicted cases were added to those already known, according to the etiological agent, and the number of cases in each group in the study sample was recalculated, obtaining new data on the prevalence of COVID-19 in this population.

**Fig 4:**
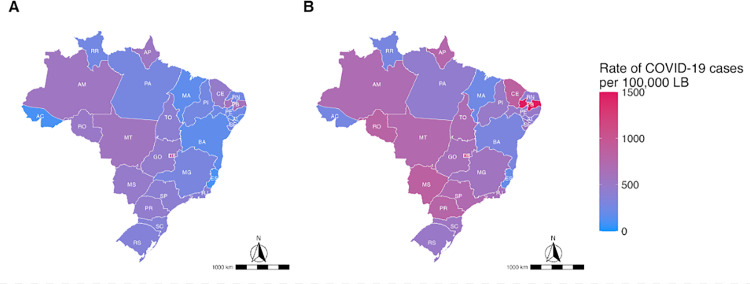
Density map of COVID-19 cases rate 4a Rate of cases officially registered as COVID-19 in Brazil; 4b Rate of COVID-19 cases after including the predicted cases.

Fig 4a shows the density map of officially registered COVID-19 cases across the national territory. At the same time, Fig 4b shows the density map of the COVID-19 rate when considering the sum of officially registered cases and the cases predicted by the XGBoost model. Fig 4 was created by the authors using the packages {geobr} in R software, which is under MIT license for free use [42] (Fig 4).

Thus, the number of SARS cases due to COVID-19 in the obstetric population was 72·1% higher (an increase of 13,799 cases) than the official reports; similarly, the number of deaths of pregnant and postpartum women due to COVID-19 increased by 19·7% (an increase of 387 deaths) compared to the officially reported numbers. The increase in case rate ranged from 11·4% to 186·2% when evaluating each of the states in Brazil, and the increase in the maternal mortality ratio ranged from 0·0% to 46·2% (S2 and S3 Tables).

It is important to note that out of the ten states with the highest rates of underreporting after the application of the prediction model, six also ranked among those with the highest increase in the maternal mortality ratio due to COVID-19 (Pernambuco, Paraiba, Esprito Santo, Alagoas, Acre, and Minas Gerais).This study constructed a predictive model for SARS etiology in pregnant and postpartum women whose notifications did not identify an etiological agent. The proposed prediction model was able to identify a 72·1% increase in cases and a 19·7% increase in maternal deaths attributed to COVID-19 in the Brazilian obstetric population from 2020 to 2021.

Accurate estimates of COVID-19 cases and deaths are a significant challenge for pandemic surveillance, especially in low- and middle-income countries, where the diagnosis is underestimated, and the disease lethality is exacerbated by a high incidence among socioeconomically disadvantaged groups and limited access to healthcare services. Socially disadvantaged populations face more difficulties in effective isolation when infected, as they reside in densely populated households with poor sanitary conditions, favoring the spread of SARS-CoV-2 [12]. A systematic review published in 2022 showed that the global number of COVID-19 deaths may have been three times higher than the official figures [43], and the region with the highest estimated underreporting of deaths caused by SARS-CoV-2 was southern Africa. When analyzed separately, the highest estimates of underreporting of deaths were seen in India, the United States, Russia, Mexico, Brazil, Indonesia and Pakistan, accounting for more than half of the number of deaths caused by COVID-19 worldwide during the period analyzed.

The research conducted in the general population by a team from the Faculty of Medicine at Universidade Federal de Minas Gerais, showed, through the analysis of medical records and exams, an 18% underreporting rate of COVID-19 deaths in Brazil, primarily due to inaccuracies in defining the underlying cause [13]. Furthermore, the death often occurred before the availability of the diagnostic test results, and a poorly defined cause or intermediate disease (garbage causes) was declared as the underlying death cause [44]. According to this research, the coronavirus identification as the probable underlying cause of death in the three capital cities (Belo Horizonte, Natal, and Salvador) analyzed was relevant, accounting for 37,163 cases (18%) only in 2020, drawing attention to the fact that the pandemic was even more severe in the country [13].

Considering that the milder cases were not prioritized for testing at the beginning of the pandemic, this fact may have contributed to this concerning rate of sub-notification in the country. The present study depicted that 5 out of 7 states with the highest rates of underreporting of COVID-19 cases were also those with the highest underreporting rates of maternal deaths, drawing attention to other factors that must be involved.

The operational difficulties in conducting diagnostic tests, a longer time frame between testing and result availability, and the alarming lack of tests during the initial phase of the pandemic may have jeopardized the correct notification of cases [45]. Despite the different rates among states, it is of utmost importance that all presented with underreporting [12]. Such disparity among reported values shows a likely difference in the policies adopted in each region.

At the pandemic’s beginning, the obstetric population was neither considered a high-risk group for developing severe COVID-19 nor a priority for diagnostic testing [15,27]. It is known that pregnant women are more susceptible to severe respiratory infections because they have a suppressed immune state and also because of the adaptive physiological changes that occur during this period, making them more intolerant to the hypoxic regime [15]. However, such a scenario changed once such a group showed a higher risk of hospitalization (337%), ICU admission (73%), and invasive ventilatory support (64%) compared to non-pregnant women [15,17,19,28,29]. Similarly, the literature demonstrated that adverse obstetric events (preeclampsia, gestational diabetes, prematurity, and fetal death) were also more prevalent in pregnant women diagnosed with COVID-19, especially those who develop severe forms of the disease. In Brazil, the Brazilian Obstetric Observatory COVID-19 (OOBr COVID-19) reported that, in the first five months of 2021, the number of deaths of pregnant and postpartum women has already exceeded the number reported throughout 2020, with a 94% increase in Maternal Mortality Ratio (MMR) [46].

During the pandemic, the country’s difficulties in accessing prenatal care and maternity services worsened. The main risk factors for severe COVID-19 in the obstetric population were related to social vulnerability, such as residing in peri-urban areas, being of black race, having lower educational levels, and the presence of comorbidities, such as obesity and gestational diabetes [24,26].

The lack of health professionals trained to offer adequate care for pregnant and postpartum women and the reduced number of ICU beds designated for this population may have also compromised the quality of care [47]. The fragility of Brazilian antenatal care became even more apparent and alarming with the increased demand for care during the pandemic.

Access to adequate care during the pandemic was crucial for the outcome of cases, showing that the lack of assistance was a determining factor for adverse outcomes in pregnant and postpartum women, leading to an increase in maternal mortality ratios during this period, especially in low- and middle-income countries [48]. Some studies showed that, in one-third of deaths among the obstetric population, patients did not have access to mechanical ventilation, and only 72% were admitted to ICU beds [30].

These weaknesses in the healthcare system may also have influenced the number of underreporting cases and, mainly, deaths from COVID-19 in pregnant and postpartum women. In Brazil, the most accurate data to assess mortality due to specific causes are obtained through the Mortality Information System (SIM), a national surveillance system developed and implemented by the Ministry of Health [49]. The availability of access to a national database (SIVEP-GRIPE), consolidated since the H1N1 pandemic in 2009, with a high number of cases of SARS in pregnant and postpartum women, provides consistency to the results obtained and to the prediction model constructed to estimate the cases and deaths that probably occurred due to COVID-19 among those for whom the etiology of SARS was not defined.

Despite the robustness of this database, a large number of “Blank” and “Unknown” responses were still observed when filling out the data on the SIVEP-GRIPE form. Thus, when creating the prediction model, it was found that the frequency of “Blank” or “Unknown” responses were higher in the COVID-19 group, although present in cases with other confirmed agents. To overcome this weakness, it was necessary to compare the groups including this variable, since failure to fill out this data could be associated with high demand, lack of training of professionals or lack of diagnostic tests, since the excess demand from the pandemic may have affected the accuracy of data entry and auditing by maternal mortality committees. Work overload for healthcare professionals, insufficient training of physicians for correct completion of death certificates, and errors in assigning the declared cause of death may have also added fragility to this system. This study was designed as a predictive model applied to the real world, that is, predicting whether a patient had COVID-19 or not based on their medical records, which commonly come with a lot of information not filled in (“Blank”) or ignored (Unknown”).

Moreover, the SIM identified a significant increase in deaths attributed to ill-defined and other natural causes in Brazil during the period, without any other explanation for the observed excess mortality. Causes such as sepsis (an increase of 30%), respiratory failure (a rise of 50%), and SARS (an increase of 381%) increased compared to data from 2017 to 2019. Conversely, deaths due to pneumonia decreased by 16%, likely because SARS was declared the cause of death instead of unspecified pneumonia in most cases [13].

This was one of the reasons why this study analyzed deaths due to SARS directly from SIVEP-Gripe rather than SIM. The mandatory notification of these cases and the clear link between death and SARS reduced potential errors in coding or underreporting due to incorrect completion of death certificates. Since these databases are public and anonymized, cross-referencing data between these two systems was not possible.

Considering the significant increase in the number of cases and deaths from COVID-19, it is important to reflect on how underreporting of these cases during the pandemic may have had negative consequences for the organization of the health system and for controlling the transmission of the virus, in addition to difficulties in providing adequate treatment, leading to an increase in serious repercussions. Such observations show the importance of knowing the real numbers to assist in implementing health policy measures, crisis coping strategies and resource planning, highlighting the need to improve data recording and continuous monitoring and the development of predictive models that can more accurately estimate the real impact of possible pandemics that may occur in the future.

Some study limitations should be disclosed. Firstly, there is a large number of “Blank” or “Unknown” responses when analyzing the data completion in the SIVEP-Gripe form. The frequency of these answers was higher in the COVID-19 group than in cases caused by other etiological agents. This variable was included in the analysis to address this limitation, as the non-completion of these data could be associated with high demand for medical care, lack of professional training, or lack of diagnostic tests. It is known that the lower availability of diagnostic tests, especially in the early stages of the pandemic, created difficulties in correctly identifying cases and deaths due to COVID-19 [13].

The study’s strong points include access to a national database consolidated since the H1N1 pandemic in 2009 and the decision to include all available variables in the model over both periods, including blank or unknown data, thus creating a realistic model.

Underreporting cases and deaths during the pandemic may have negatively affected the healthcare system organization and the virus transmission control, leading to difficulties in adequate treatment and severe repercussions. These observations underscore the importance of knowing the actual numbers of cases to assist in implementing health policy measures, crisis management strategies, and resource planning, highlighting the need for improved data recording, continuous monitoring, and the development of predictive models that can estimate more accurately the actual impacts of any future pandemics [50].

## Conclusion

This study evaluated the number of underreported cases and deaths due to SARS in pregnant and postpartum women in Brazil and its states. The results indicate that, during the pandemic, the number of COVID-19 cases and deaths in the obstetric population was much higher than documented by authorities, suggesting that the disease’s impact on the maternal mortality ratio during the period was even more significant than reported.

## Supporting information

S1 Table Other hyperparameters adopted in the XGBoost model. (DOCX)

S2Table Sociodemographic Characteristics, clinical presentation, and presence of comorbidities among Pregnant and Postpartum Women with SARS according to the etiological agent, either confirmed or predicted. (DOCX)

S3 Table COVID-19 case rate by Brazilian sates recalculated after adding confirmed COVID-19 cases to predicted COVID-19 cases using the XGBoost prediction model. (DOCX)

S4 Table COVID-19 death rate by Brazilian sates recalculated after adding confirmed COVID-19 cases to predicted COVID-19 cases using the XGBoost prediction model. (DOCX)
