# Supplementary material for: Unveiling the hidden burden of COVID-19 in Brazil’s obstetric population with severe acute respiratory syndrome: A machine learning model
Source: PLoS One. 2025 Aug 22;20(8):e0330375. doi: 10.1371/journal.pone.0330375 (PMC12373234; doi:10.1371/journal.pone.0330375)
Supplement: S1 Table — (DOCX) [file pone.0330375.s001.docx]

**S1 Table: Other hyperparameters adopted in the machine learning models**

| **Model** | **Hyperparameter** | **Value** |
| --- | --- | --- |
| Lasso Lgistic Regression | *λ* (penalty) | 0.0000580 |
| K-nearest Neighbors | *k* (neighbors) | 14 |
| Classification Trees | cost_complexity | 0.0001 |
|  | tree_depth | 15 |
|  | min_n | 27 |
| Random Forests | mtry | 5 |
| Bagging | cost_complexity | 0 |
|  | min_n | 2 |
| RBF SVM | cost | 5 |
|  | rbf_sigma | 0.25 |
| Polynomial SVM | cost | 0.5 |
|  | degree | 1 |
