# Supplementary material for: Unveiling the hidden burden of COVID-19 in Brazil’s obstetric population with severe acute respiratory syndrome: A machine learning model
Source: PLoS One. 2025 Aug 22;20(8):e0330375. doi: 10.1371/journal.pone.0330375 (PMC12373234; doi:10.1371/journal.pone.0330375)
Supplement: S2Table — (DOCX) [file pone.0330375.s002.docx]

S2 Table: Sociodemographic Characteristics, clinical presentation, and presence of comorbidities among Pregnant and Postpartum Women with SARS according to the etiological agent, either confirmed or predicted

| Variable | Confirmed COVID-19  N=19138 | Predicted COVID-19  N=13799 | Other confirmed agents N=2233 | Predicted Other agents  N=179 |
| --- | --- | --- | --- | --- |
| SARS notifications/day; mean ± sd | 47.60 ± 25.89 | 24.39 ± 7.65 | 9.41 ± 14.36 | 6.66 ± 6.77 |
| Age (years); mean ± sd | 29.86 ± 7.20 | 27.47 ± 7.43 | 27.45 ± 7.05 | 25.93 ± 7.94 |
| Race, N (%)  White  Non-white  Blank  Ignored | 6649 (34.7%) | 4015 (29.1%) | 1011 (45.3%) | 72 (40.2%) |
|  | 9749 (50.9%) | 7913 (57.3%) | 981 (43.9%) | 88 (49.2%) |
|  | 262 (1.4%) | 224 (1.6%) | 53 (2.3%) | 1 (0.6%) |
|  | 2478 (12.9%) | 1647 (11.9%) | 189  (8.5%) | 18 (10.1%) |
| Education level, N (%)  Up to elementary  High school  Higher education  Blank  Ignored | 2317 (12.1%) | 2172 (15.7%) | 434 (19.4%) | 36 (20.1%) |
|  | 4445 (23.2%) | 3274 (23.7%) | 715 (32.0%) | 41 (22.9%) |
|  | 1557 (8.1%) | 666 (4.8%) | 245 (11.0%) | 16 (8.9%) |
|  | 5664 (29.6%) | 3792 (27.5%) | 301 (13.5%) | 44 (24.6%) |
|  | 5155 (26.9%) | 3895 (28.2%) | 538 (24.1%) | 42 (23.5%) |
| Influenza vaccination, N (%)  Yes  No  Blank  Ignored | 2781 (14.5%) | 2618 (19.0%) | 609 (27.3%) | 36 (20.1%) |
|  | 5996 (31.3%) | 4340 (31.5%) | 1156 (51.8%) | 92 (51.4%) |
|  | 3850 (20.1%) | 2669 (19.3%) | 124 (5.6%) | 19 (10.6%) |
|  | 6511 (34.0%) | 4172 (30.2%) | 344 (15.4%) | 32 (17.9%) |
| Gestational stage, N (%)  First trimester  Second trimester  Third trimester  Postpartum  Ignored | 1394 (7.3%) | 1357 (9.8%) | 366 (16.4%) | 27 (15.1%) |
|  | 4014 (21.0%) | 2799 (20.3%) | 701 (31.4%) | 67 (37.4%) |
|  | 9410 (49.2%) | 5885 (42.6%) | 920 (41.2%) | 52 (29.1%) |
|  | 3540 (18.5%) | 3252 (23.6%) | 204 (9.1%) | 25 (14.0%) |
|  | 780 (4.1%) | 506 (3.7%) | 42 (1.9%) | 8 (4.5%) |
| Fever  Yes  No  Blank  Ignored | 10129 (52.9%) | 5914 (42.9%) | 1803 (80.7%) | 134 (74.9%) |
|  | 6318 (33.0%) | 5645 (40.9%) | 378 (16.9%) | 40 (22.3%) |
|  | 2519 (13.2%) | 2109 (15.3%) | 38 (1.7%) | 5 (2.8%) |
|  | 172 (0.9%) | 131 (0.9%) | 14 (0.6%) | 0 (0.0%) |
| Cough  Yes  No  Blank  Ignored | 12882 (67.3%) | 8206 (59.5%) | 2022 (90.6%) | 156 (87.2%) |
|  | 4273 (22.3%) | 3964 (28.7%) | 173 (7.7%) | 19 (10.6%) |
|  | 1838 (9.6%) | 1523 (11.0%) | 32 (1.4%) | 4 (2.2%) |
|  | 145 (0.8%) | 106 (0.8%) | 6 (0.3%) | 0 (0.0%) |
| Sore throat  Yes  No  Blank  Ignored | 3917 (20.5%) | 2790 (20.2%) | 913 (40.9%) | 71 (39.7%) |
|  | 10683 (55.8%) | 7868 (57.0%) | 1191 (53.3%) | 96 (53.6%) |
|  | 4251 (22.2%) | 2941 (21.3%) | 77 (3.4%) | 10 (5.6%) |
|  | 287 (1.5%) | 200 (1.4%) | 52 (2.3%) | 2 (1.1%) |
| Dyspnea  Yes  No  Blank  Ignored | 10721 (56.0%) | 5575 (40.4%) | 1438 (64.4%) | 125 (69.8%) |
|  | 5884 (30.7%) | 5839 (42.3%) | 719 (32.2%) | 52 (29.1%) |
|  | 2391 (12.5%) | 2242 (16.2%) | 56 (2.5%) | 2 (1.1%) |
|  | 142 (0.7%) | 143 (1.0%) | 20 (0.9%) | 0 (0.0%) |
| Respiratory discomfort  Yes  No  Blank  Ignored | 8424 (44.0%) | 4786 (34.7%) | 1495 (67.0%) | 126 (70.4%) |
|  | 7267 (38.0%) | 6312 (45.7%) | 643 (28.8%) | 50 (27.9%) |
|  | 3239 (16.9%) | 2541 (18.4%) | 69 (3.1%) | 3 (1.7%) |
|  | 208 (1.1%) | 160 (1.2%) | 26 (1.2%) | 0 (0.0%) |
| O2 saturation < 95%  Yes  No  Blank  Ignored | 7187 (37.6%) | 3017 (21.9%) | 675 (30.2%) | 65 (36.3%) |
|  | 8315 (43.4%) | 7706 (55.8%) | 1388 (62.2%) | 101 (56.4%) |
|  | 3391 (17.7%) | 2878 (20.9%) | 93 (4.2%) | 10 (5.6%) |
|  | 245 (1.3%) | 198 (1.4%) | 77 (3.4%) | 3 (1.7%) |
| Diarrhea  Yes  No  Blank  Ignored | 1847 (9.7%) | 1113 (8.1%) | 54 (2.4%) | 7 (3.9%) |
|  | 12270 (64.1%) | 9141 (66.2%) | 784 (35.1%) | 145 (81.0%) |
|  | 4746 (24.8%) | 3337 (24.2%) | 1369 (61.3%) | 25 (14.0%) |
|  | 275 (1.4%) | 208 (1.5%) | 26 (1.2%) | 2 (1.1%) |
| Heart disease  Yes  No  Blank  Ignored | 1157 (6.0%) | 756 (5.5%) | 54 (2.4%) | 4 (2.2%) |
|  | 5945 (31.1%) | 4758 (34.5%) | 1517 (67.9%) | 80 (44.7%) |
|  | 11899 (62.2%) | 8175 (59.2%) | 617 (27.6%) | 92 (51.4%) |
|  | 137 (0.7%) | 110 (0.8%) | 45 (2.0%) | 3 (1.7%) |
| Chronic lung disease  Yes  No  Blank  Ignored | 117 (0.6%) | 144 (1.0%) | 115 (5.2%) | 7 (3.9%) |
|  | 6660 (34.8%) | 5137 (37.2%) | 1452 (65.0%) | 76 (42.5%) |
|  | 12112 (63.8%) | 8403 (60.9%) | 620 (27.8%) | 93 (52.0%) |
|  | 149 (0.8%) | 115 (0.8%) | 46 (2.1%) | 3 (1.7%) |
| Chronic kidney disease  Yes  No  Blank  Ignored | 124 (0.6%) | 90 (0.7%) | 8 (0.4%) | 3 (1.7%) |
|  | 6607 (34.5%) | 5136 (37.2%) | 1553 (69.5%) | 78 (43.6%) |
|  | 12263 (64.1%) | 8452 (61.3%) | 627 (28.1%) | 95 (53.1%) |
|  | 144 (0.8%) | 121 (0.9%) | 45 (2.0%) | 3 (1.7%) |
| Obesity, N (%)  Yes  No  Blank  Ignored | 1183 (6.2%) | 467 (3.4%) | 61 (2.7%) | 4 (2.2%) |
|  | 5868 (30.7%) | 4866 (35.3%) | 1489 (66.7%) | 75 (41.9%) |
|  | 11922 (62.3%) | 8336 (60.4%) | 639 (28.6%) | 98 (54.7%) |
|  | 165 (0.9%) | 130 (0.9%) | 44 (2.0%) | 2 (1.1%) |

COVID-19: *Coronavirus Disease 2019;* sd: standard deviation; N: number; % percentage.
