# Supplementary material for: Unveiling the hidden burden of COVID-19 in Brazil’s obstetric population with severe acute respiratory syndrome: A machine learning model
Source: PLoS One. 2025 Aug 22;20(8):e0330375. doi: 10.1371/journal.pone.0330375 (PMC12373234; doi:10.1371/journal.pone.0330375)
Supplement: S3 Table — (DOCX) [file pone.0330375.s003.docx]

S3 Table:    COVID-19 case rate by Brazilian sates recalculated after adding confirmed COVID-19 cases to predicted COVID-19 cases using the XGBoost prediction model.

| Brazilian state | Number of COVID-19 cases (confirmed) | Number of COVID-19 cases (confirmed + predicted) | COVID-19 case rate (confirmed) | COVID-19 case rate (confirmed + predicted) | Case rate* increase (percentage) |
| --- | --- | --- | --- | --- | --- |
| Rondônia | 258 | 420 | 503.5 | 819.7 | 62.8 |
| Acre | 29 | 83 | 94.0 | 269.1 | 186.2 |
| Amazonas | 850 | 1074 | 551.6 | 697.0 | 26.4 |
| Roraima | 70 | 78 | 253.1 | 282.0 | 11.4 |
| Pará | 744 | 1209 | 275.5 | 447.7 | 62.5 |
| Amapá | 156 | 226 | 526.6 | 762.8 | 44.9 |
| Tocantins | 190 | 317 | 400.2 | 667.7 | 66.8 |
| Maranhão | 368 | 509 | 171.4 | 237.0 | 38.3 |
| Piauí | 236 | 444 | 258.8 | 486.8 | 88.1 |
| Ceará | 1111 | 2083 | 458.8 | 860.1 | 87.5 |
| Rio Grande do Norte | 227 | 378 | 261.0 | 434.7 | 66.5 |
| Paraíba | 680 | 1653 | 604.8 | 1470.3 | 143.1 |
| Pernambuco | 581 | 1221 | 228.1 | 479.4 | 110.2 |
| Alagoas | 179 | 398 | 184.3 | 409.7 | 122.3 |
| Sergipe | 164 | 333 | 260.4 | 528.7 | 103.0 |
| Bahia | 635 | 1129 | 169.6 | 301.5 | 77.8 |
| Minas Gerais | 1428 | 2806 | 291.8 | 573.4 | 96.5 |
| Espírito Santo | 107 | 215 | 100.7 | 202.3 | 100.9 |
| Rio de Janeiro | 1426 | 2435 | 366.6 | 626.0 | 70.8 |
| São Paulo | 4343 | 7712 | 403.0 | 715.7 | 77.6 |
| Paraná | 1279 | 2295 | 443.7 | 796.1 | 79.4 |
| Santa Catarina | 687 | 1060 | 353.4 | 545.2 | 54.3 |
| Rio Grande do Sul | 888 | 1284 | 348.0 | 503.2 | 44.6 |
| Mato Grosso do Sul | 393 | 713 | 470.8 | 854.1 | 81.4 |
| Mato Grosso | 649 | 848 | 564.9 | 738.2 | 30.7 |
| Goiás | 826 | 1145 | 449.6 | 623.2 | 38.6 |
| Distrito Federal | 634 | 869 | 819.2 | 1122.8 | 37.1 |

*Rate by 100,000 live births
