# Supplementary material for: Unveiling the hidden burden of COVID-19 in Brazil’s obstetric population with severe acute respiratory syndrome: A machine learning model
Source: PLoS One. 2025 Aug 22;20(8):e0330375. doi: 10.1371/journal.pone.0330375 (PMC12373234; doi:10.1371/journal.pone.0330375)
Supplement: S4 Table — (DOCX) [file pone.0330375.s004.docx]

S4 Table: COVID-19 death rate by Brazilian sates recalculated after adding confirmed COVID-19 cases to predicted COVID-19 cases using the XGBoost prediction model.

| Brazilian state | Number of COVID-19 deaths (confirmed) | Number of COVID-19 deaths (confirmed + predicted) | MMR by COVID-19 (confirmed) | MMR by COVID-19 (confirmed + predicted) | MMR* increase (percentage) |
| --- | --- | --- | --- | --- | --- |
| Rondônia | 35 | 36 | 68.3 | 70.3 | 2.9 |
| Acre | 8 | 10 | 25.9 | 32.4 | 25.0 |
| Amazonas | 81 | 88 | 52.6 | 57.1 | 8.6 |
| Roraima | 30 | 30 | 108.5 | 108.5 | 0.0 |
| Pará | 79 | 105 | 29.3 | 38.9 | 32.9 |
| Amapá | 13 | 14 | 43.9 | 47.3 | 7.7 |
| Tocantins | 25 | 28 | 52.7 | 59.0 | 12.0 |
| Maranhão | 76 | 86 | 35.4 | 40.0 | 13.2 |
| Piauí | 28 | 31 | 30.7 | 34.0 | 10.7 |
| Ceará | 81 | 96 | 33.4 | 39.6 | 18.5 |
| Rio Grande do Norte | 53 | 61 | 60.9 | 70.1 | 15.1 |
| Paraíba | 45 | 65 | 40.0 | 57.8 | 44.4 |
| Pernambuco | 39 | 57 | 15.3 | 22.4 | 46.2 |
| Alagoas | 25 | 32 | 25.7 | 32.9 | 28.0 |
| Sergipe | 15 | 17 | 23.8 | 27.0 | 13.3 |
| Bahia | 92 | 124 | 24.6 | 33.1 | 34.8 |
| Minas Gerais | 149 | 183 | 30.4 | 37.4 | 22.8 |
| Espírito Santo | 25 | 32 | 23.5 | 30.1 | 28.0 |
| Rio de Janeiro | 252 | 285 | 64.8 | 73.3 | 13.1 |
| São Paulo | 355 | 437 | 32.9 | 40.6 | 23.1 |
| Paraná | 127 | 149 | 44.1 | 51.7 | 17.3 |
| Santa Catarina | 52 | 58 | 26.7 | 29.8 | 11.5 |
| Rio Grande do Sul | 71 | 85 | 27.8 | 33.3 | 19.7 |
| Mato Grosso do Sul | 33 | 38 | 39.5 | 45.5 | 15.2 |
| Mato Grosso | 48 | 54 | 41.8 | 47.0 | 12.5 |
| Goiás | 107 | 126 | 58.2 | 68.6 | 17.8 |
| Distrito Federal | 22 | 26 | 28.4 | 33.6 | 18.2 |

*Rate by 100,000 live births
